# The Role of Environmental Risk Factors on the Development of Childhood Allergic Rhinitis

**DOI:** 10.3390/children8080708

**Published:** 2021-08-17

**Authors:** Allison C. Wu, Amber Dahlin, Alberta L. Wang

**Affiliations:** Channing Division of Network Medicine, Mass General Brigham, Boston, MA 02115, USA; amber.dahlin@channing.harvard.edu (A.D.); alberta.wang@channing.harvard.edu (A.L.W.)

**Keywords:** allergic rhinitis, child, pediatrics, air pollution, allergens, traffic related air pollution, tobacco smoke, climate change, viruses, greenness, therapeutics, prevention

## Abstract

Environmental factors play an important role in the development and exacerbation of allergic rhinitis (AR) in childhood. Indoor air pollution, such as house dust mites and secondhand smoke, can significantly increase the onset of AR, while pet dander may affect the exacerbation of AR symptoms in children. Furthermore, traffic related air pollution and pollen are outdoor air pollutants that can affect immune competency and airway responsiveness, increasing the risk of AR in children. Climate change has increased AR in children, as growth patterns of allergenic species have changed, resulting in longer pollen seasons. More extreme and frequent weather events also contribute to the deterioration of indoor air quality due to climate change. Additionally, viruses provoke respiratory tract infections, worsening the symptoms of AR, while viral infections alter the immune system. Although viruses and pollution influence development and exacerbation of AR, a variety of treatment and prevention options are available for AR patients. The protective influence of vegetation (greenness) is heavily associated with air pollution mitigation, relieving AR exacerbations, while the use of air filters can reduce allergic triggers. Oral antihistamines and intranasal corticosteroids are common pharmacotherapy for AR symptoms. In this review, we discuss the environmental risk factors for AR and summarize treatment strategies for preventing and managing AR in children.

## 1. Introduction

A global health problem with significant economic burden, allergic rhinitis (AR) has established itself as the most common chronic allergic disease and affects approximately 40% of the population worldwide [1,2,3]. In the United States AR is the most common chronic illness in children [4,5,6]. Children with AR have decreased productivity at school and lower grades [7]. AR is a chronic inflammatory disease in the upper airways that is induced by an abnormal immunological response to airborne antigens [8]. It is an immunoglobulin-E (IgE) mediated type 1 hypersensitivity illness triggered by a wide array of environmental allergens such as pollen, mold, and dust [9]. AR differs from nonallergic rhinitis as AR includes allergic sensitization or symptoms during an exposure, whereas nonallergic rhinitis is not associated with IgE-mediated sensitization [10]. One study suggested that AR is associated with irreversible nasal airway obstruction and eosinophilic inflammation, while nonallergic rhinitis is associated with no change in nasal airway patency and less nasal mucosal eosinophilia than AR [10].

The most common symptoms of AR include nasal congestion, nasal pruritus, sneezing, and runny nose, leading to impairment in daily activities, sleep habits, cognitive function, work and school productivity, and overall quality of life [8,11,12]. Children with AR are more likely to have asthma, allergic conjunctivitis, rhinosinusitis, nasal polyposis, and otitis media [8]. Currently, studies evaluating the association between environmental risk factors with childhood AR have not produced definitive conclusions because of varying in study designs and length of exposures and the complex composition of pollutants [9]. Children may experience greater exposure to environmental allergens and pollutants and increased morbidity from AR due to higher rates of oxygen consumption per unit body weight and immature respiratory and immunological systems, respectively [13]. Furthermore, children play outdoors for periods of time and thereby have increased exposure to outdoor environmental factors [13]. While the development of AR has genetic contributions, this article focuses on the role of environmental risk factors, including indoor and outdoor air pollution and viruses [14].

Pollution, considered the largest single environmental risk for health, is the introduction of harmful substances into the environment, contaminating air, soil, or water with chemical substances or energy [15]. Pollution is often associated with AR, allergic sensitization, and autoimmunity and can have detrimental health effects involving the immune system [16]. When atopic individuals are exposed to allergens and pollutants, they develop specific IgE antibodies that reside on the surfaces of mast cells and other immune cells [9]. With additional exposure to allergens and pollutants, histamines, arachidonic acid metabolites, and other inflammatory mediators are released from mast cells, resulting in sneezing, nasal congestion, and other common AR symptoms [17]. Pollutants can provoke the nasal mucosa, allowing the release of mediators of allergic inflammation and increasing nasal hyperreactivity [9]. Both outdoor air pollution, such as traffic related air pollution (TRAP), and indoor air pollution, including pet dander, molds, and tobacco smoke, contribute to the development of AR in children [18].

## 2. Indoor Exposures

Indoor air pollution including tobacco smoke, indoor allergens (e.g., dust mites, pet dander, molds), and other pollutants (e.g., cleaning chemicals) all contribute to the development of AR and the aggravation of symptoms [9]. Individuals in western countries spend the vast majority of their time indoors, and more than 90% of the population live in places where air quality does not meet World Health Organization (WHO) standards, leading to increased rates of AR in children [19]. Furthermore, during the Health Effects of School Environment (HESE) project, it was found that 78% of children attending schools in Norway, Sweden, Denmark, France, and Italy are exposed to high levels of inhalable particulate matter with a diameter of 10 micrograms (PM_10_), and 66% are exposed to carbon dioxide (CO_2_) over 1000 ppm [20]. It was also demonstrated that concentrations of pollutants from industrial emissions were higher in urban schools than rural schools, indicating that the quality of indoor air is diminished from the diffusion of outdoor pollutants [21].

Common indoor pollutants include nitrogen dioxide (NO_2_), carbon monoxide (CO), and volatile organic compounds (VOC) [22]. Gas fueled cooking and heating appliances produce NO_2_, while VOCs are frequently released by consumer products such as cleaning chemicals, cosmetics, and air fresheners [23]. Evidence suggests that exposure to VOCs is associated with the development of AR and aggravation of AR symptoms [18]. Additionally, in a recent meta-analysis, sanitation workers exposed to high amounts of chemicals and pollutants reported higher inflammatory and respiratory symptoms than non-sanitation workers [24].

### 2.1. Tobacco Smoke

Several studies have evaluated whether tobacco smoke exposure could cause AR, as tobacco smoke has already been demonstrated to play a major negative impact on global public health [6,25]. The prevalence of smoke exposure is very high worldwide, and approximately 78% of children in Europe are exposed to secondhand smoke [6]. Moreover, 14% of adolescents aged 13 to 15 years are active smokers, and 25% have had their first cigarette by the age of 10 years [6]. Tobacco exposure induces inflammatory and immune responses [26].

A meta-analysis found that children actively smoking had an increased risk of AR, while those exposed to second-hand smoke (passive smoking) had a much larger increased risk for AR [6]. In a French epidemiological study, children actively smoking were almost three times more likely to experience symptoms of AR than non-smoking children [19]. Maternal smoking was also found to contribute to AR development in the child. Because children’s respiratory, nervous, and immune systems are not yet mature, children are more susceptible to the health effects of smoking [6,27]. Tobacco smoke can upregulate airway mucus production and impair mucociliary clearance, inducing low-grade inflammation within the lungs [28]. Small changes in micro-environmental conditions can also affect local microbiota within the respiratory system and promote airway remodeling [18]. Exposure to tobacco smoke may also increase aeroallergen sensitization and exacerbate AR symptoms [19]. A birth cohort study of over 4000 children followed for 16 years found that exposure to secondhand smoke during infancy was associated with a 1.18 increased risk of rhinitis up to 16 years of age, but exposure to secondhand smoke throughout childhood was not associated with the development of rhinitis, suggesting an early window of susceptibility to SHS [29]. Moreover, the Irish International Study of Asthma and Allergies in Childhood (ISAAC) study found that children ages 13–14 years who were exposed to secondhand smoke were significantly more likely to have symptoms of AR with an adjusted OR 1.35 (95% CI 1.08–1.70) [30]. In a 2020 study of university students and staff, tobacco smoke appeared to increase nasal resistance and worsen inflammation in AR patients [19]. Additionally, another study found that tobacco causes an increase in IL-33, a driver of Th2-oriented cytokines necessary to activate inflammatory cells and regulate immunity [31].

### 2.2. Indoor Allergens

House dust mites (HDM), molds, and pet dander are common examples of indoor allergens [18]. Indoor allergens cause more severe allergy symptoms than outdoor allergens [18]. Longitudinal data collected between 2006 and 2017 suggest that exposure to molds is tightly linked to the development and exacerbation of symptoms of AR in children [32].

A meta-analysis suggests that the risk for AR is significantly increased with exposure to home dampness and mold [33]. Dampness is present in around 15% of households, which can lead to the development of mold and infestation of cockroaches [16]. Because mold can stimulate inflammation in airways due to metabolites like glucans and mycotoxins, mold odor and visible mold increase the risk for development of AR [18,33]. Specific molds such as Penicillium, Alternaria, and Cladosporium are known to be associated with the development of AR [9]. Moreover, water damaged environments can be hazardous because they release mycotoxins, leading to respiratory tract disease, while fungal colonies can release antigens and toxins through fungal fragments [9].

Additionally, HDM are perpetual triggers for airway allergy [16]. Data have shown that 1 to 2 percent of the global population is sensitized to HDM [34]. HDM can be found in household dust, mattresses, pillows, bed linens, floor carpeting, upholstered furniture, and unwashed clothing [9]. Larger populations of HDM will be found in places of higher humidity [34]. Although not always a linear correlation, AR development and exacerbation are linked to HDM exposure [34]. Sensitization to HDM in newborns and school children can promote AR later in life [9,16]. When following newborn children for the first 3 years of life, the development of AR increased when exposed to major HDM allergens, as shown by the German Multicentre Allergy Study [34].

Animal-derived allergens are one of the most common sources of aeroallergens and triggers for AR with over 15% of the world’s population sensitized to furred animals with a high rate of cross-reactivity within species [18,35]. Cats, dogs, mice, and other animals produce allergens that are often prevalent in proximity to metropolitan areas, and these allergens can linger after the removal of the animals or pets and continue to cause symptoms [35]. Cat dander, specifically, can linger 6–9 months after removal of the cat [36]. Nevertheless, some studies have shown that children exposed to cat and dog dander have a lower prevalence of allergic sensitization, suggesting that early exposure to allergens could actually be protective against sensitization [37]. Adults, however, have consistently had a significantly higher prevalence of AR symptoms when exposed to dog and cat dander [37].

## 3. Outdoor Exposures

The major outdoor exposures that play a role in AR include outdoor air pollution and pollen. Outdoor air pollution caused by traffic, which produces ozone (O_3_), NO_2_, sulfur dioxide (SO_2_), and particulate matter (PM), contributes to AR development and symptoms in children [6,18]. Air pollution affects human health by making some plants more allergenic while contributing to global warming [18]. Climate change has critical influences on airborne allergens and outdoor pollution, while exposure to greenness appears to protect against AR.

### 3.1. Outdoor Air Pollution

Outdoor air pollution is known to affect immune competency and airway responsiveness, increasing the risk of AR in children [8]. Because airways are one of the major parts of the body that are exposed to the environment, the measure of all environmental exposures capable of influencing human health, also known as the exposome, can greatly affect the homeostasis of respiratory tract [18]. When changes occur in the local environment of the respiratory tract, which houses a variety of bacteria, viruses, and fungi, long lasting bacterial dysbiosis may result [18]. Low O_3_ concentrations and NO_2_ can cause inflammation of the human nasal mucosa in cell studies, and atrophy or pre-existing inflammation increases the sensitivity to these gases, increasing susceptibility to AR [8]. O_3_ can also trigger the cellular membrane of nasal epithelia to release cytokines and arachidonic acid metabolites, upregulating local inflammation [9]. Furthermore, exposure to PM can lead to oxidative stress, airway hyperresponsiveness, and airway remodeling [8]. In children living close to industrial sources, SO_2_ released from petroleum is associated with acute respiratory symptoms [9].

The exposome, which accounts simultaneously for all internal and external exposures, is associated with the development of and exacerbations from AR [38]. The external exposome includes exposure to (1) external environmental factors, such as in utero smoking, bio-contaminants (e.g., viruses), air pollutants, diet, allergens (e.g., pollens, molds, pets), and consumer products and (2) nonspecific general exposures, such as climate, biodiversity (e.g., greenspace), and social dimension, and mobility [38]. The internal exposome is specific to each subject and includes transcriptomics, adductomics, metabolomics, and proteomics. Together, the external and internal exposomes contribute to the risk of AR and other allergic diseases [38].

The responses that individuals with AR have to allergens differ from healthy individuals [9]. For example, individuals with seasonal AR experience higher levels of nasal congestion than individuals without AR when exposed to chlorine gas in a controlled chamber [9]. Furthermore, allergic reactions can be aggravated when small sized PM enter the upper respiratory tract and mucosal barrier because PM can act as allergen carriers [8,39].

### 3.2. Traffic Related Air Pollution (TRAP)

TRAP is the combination of black carbon from diesel exhaust, nitrous oxides from general traffic, carbon monoxide from petrol exhaust, zinc from automobile brakes, and copper from tires [9,40]. In the Sydney metropolitan area, a study examined the response of primary murine and human airway epithelial cells (AECs) to TRAP or ambient PM and demonstrated that ambient PM_10_ caused stronger secretion of IL-6 and CXCL1 by AECs [18]. In addition, a study of 2598 children suggested that TRAP exposure in utero and the first year of life may lead to the development of AR in preschool children [3]. In another study following children exposed to TRAP at birth until age 4 years, it was found that those exposed to diesel exhaust particles at age 1 years were sensitized to aeroallergens at ages 2 and 3 years [41]. Some studies with close proximity to traffic pollution have demonstrated an increased risk of exacerbation of respiratory symptoms; however, other long-term studies have failed to demonstrate any positive association [42].

### 3.3. Pollen

Birch pollen is the most common tree pollen in Northern and Central Europe and is prevalent throughout the Northern hemisphere [43]. Birch pollen is a major cause of AR, and the prevalence of sensitization to birch pollen has risen recently, contributing to prolonged AR symptoms due to cross-reactivity with other plant allergens [43]. Alder, hazelnut, hornbeam, oak, chestnut, and beech trees are all associated with AR, likely because these trees are part of the Fagles and Betulaceae families, similar to birch [44]. Additionally, approximately 70% of individuals with birth pollen allergies experience pollen food syndrome, where ingestion of raw fruits, vegetables, roots, and nuts lead to localized IgE-mediated symptoms due to cross-reactivity between aeroallergens and food allergens [45,46]. Multiple studies suggest that higher surrounding pollen concentration is associated with increased AR symptoms severity [47,48].

Studies have inconsistently demonstrated that urban dwellings pose a higher risk of AR than suburban dwellings because of the loss of biodiversity with urbanization [18,38]. With high CO_2_ concentrations in urban areas, plants such as ragweed can flower earlier, resulting in longer pollen seasons and the production of more pollen [49]. Pollen grains and fungal spores also contain bioactive elements that can exert inflammatory and allergic effects, increasing rates of AR in children [50]. Additionally, in a study with 1360 Italian children, the average age of onset of pollen-induced AR was 5.3 years (SD ± 2.8), and the average disease duration was 5.2 years (SD ± 3.3) [11]. In this study, 6.2% of the children had pollen monosensitization, while 84.9% were sensitized to at least three pollen extracts, most commonly timothy grass or olive tree pollen [11]. The majority of the children had AR symptoms during the spring; however, some patients reported symptoms during the fall as well [11]. Furthermore, in a Swedish study of 764 children, IgE sensitization to Bet v 1, the major birch tree allergen, in early childhood, was a predictor of AR by the age of 16 years [51]. A German study showed that birch trees exposed to higher concentrations of O_3_ produced more birch allergen and pollen associated lipid mediators (PALMs) per pollen grain than the ozone free trees [52]. PALMs activate Th2 cells and promote IgE synthesis [53]. Moreover, on skin prick tests in AR patients, extract from the O_3_ exposed trees demonstrated a larger wheal diameter than the less exposed trees [18].

### 3.4. Climate Change

Climate change, long-term shifts in weather patterns including changes in temperature, has an impact on respiratory allergies because changes in meteorological variables affect the prevalence of airborne allergens and ambient pollution [54]. Currently, as environmental conditions are altered due to climate change, AR in children is increasing [8]. Mathematical simulation studies suggest that climate change will increase the severity of AR by up to 60% [55]. The majority of today’s global energy is generated by burning fossil fuels, releasing CO_2_, methane, black carbon, nitrogen oxides, and sulfate aerosols into the environment [56,57,58]. Greenhouse gases keep the earth warm by absorbing the sun’s energy and redirecting it back to the earth’s surface; however, an overabundance of the gases traps an excessive amount of heat in the atmosphere, resulting in global warming [59]. Climate change also influences the amount and type of pollutants in the air, which interact with altered levels of aeroallergens [18].

The seasonality and severity of AR are affected by the growth patterns of different allergenic species, and global warming and air pollution are known to affect these patterns [18]. Global warming changes local vegetation patterns and increases the growth rate of plants, increasing airborne pollen concentrations [60]. Different North American and European studies have interlinked climate change with the increased duration of the ragweed pollen season [49]. Birch pollen levels have risen over the past few decades due to climate change [43]. Additionally, climate change has contributed to alterations in the geographical spread of plants [61]. For example, ragweed is a native North American plant but is now invading several European areas [61]. The colonization of these new areas by novel species is suspected to induce respiratory symptoms by de novo sensitization and cross-reactivity with pre-existing species [62]. Moreover, climate change causes more frequent extreme climate events, including intensive rain and flooding, which can lead to the deterioration of indoor air quality by promoting dampness and mold [63]. Heavy rainfall and thunderstorms can also increase the atmospheric concentration of allergenic particles, increasing airway inflammation [63]. Climate change is implicated in the worsening of symptoms and increased frequency of use of medication to control AR symptoms [64].

### 3.5. Greenness

Greenness, areas with increased presence of trees and green spaces, helps mitigate air pollution [65]. Greenness can protect individuals by removing PM, CO, CO_2_, NO_2_, O_3_, and SO_2_, from air pollution [66]. During a plant’s life cycle, 0.5 to 6 tons of excess atmospheric CO_2_ can be consumed [67]. In 2008, a Spanish ecological study found that trees and shrubs removed 305.6 tons of pollutants (166 tons of PM_10_, 73 tons of O_3_, 55 tons of NO_2_, 7 tons of SO_2_ and 6 tons of CO) [68]. Species with excellent removal of outdoor air pollution include elm trees, common ash, wild lime trees, verrucous birch, curly maple, and hackberry [69]. The Normalized Difference Vegetation Index (NDVI) uses different intensities of reflected and near-infrared light to estimate the density of chlorophyll containing vegetation [70]. NDVI ranges from −1 to +1, with positive values indicating high greenness [70]. Healthy vegetation reflects more near-infrared and green light, while it absorbs more red and blue light [70]. Overall, children residing in places with less greenness have higher prevalence of AR [70].

Although findings from studies of greenness are inconsistent, greater greenness may prevent AR and its symptoms [66,71]. A possible contributing factor is mountain soil, due to the diverse microbial communities it contains that confer a protective effect against AR in children in settings with soil exposure [72]. Additionally, having a garden at home may be associated with a decreased likelihood of AR because gardens provide a nearby bio-diverse neighborhood and rich density of outdoor microbiota [73]. A larger distance to nature was associated with more AR symptoms likely related to higher levels of NO_2_ based on an Austrian study [74]. Furthermore, greater NDVI and tree cover (surfaces covered by woody vegetation taller than 5 m) were marginally associated with fewer AR symptoms, as NO_2_ levels are lower [74]. A study that followed adults sensitized to *Betulaceae* pollen during tree pollen season assessed exposure to green space and allergenic trees while tracking daily symptom severity scores [75]. The study concluded that exposure to green space may decrease tree pollen allergy symptom severity but only when the density of allergenic trees is low [75].

## 4. Viruses

Viruses, such as adenoviruses, coronaviruses, influenza viruses, parainfluenza viruses, rhinoviruses, and respiratory syncytial viruses, trigger upper respiratory infections (URI), which are the most common infections in humans [76]. Viral infections can lead to both the development and exacerbation of AR [76]. Viral infections activate the immune system and contribute to both an enhancing effect, exacerbating symptoms of AR, and protective effect, decreasing the chance of allergic development [77]. Furthermore, symptoms of a viral respiratory illness can be more pronounced in individuals with a history of AR [76].

Rhinovirus infection has been shown to increase early and late phase reactions to allergens and, thus, may promote the development of allergic airway disease [76]. In a study of 38 individuals with and without seasonal AR, subjects with AR had an earlier onset of sneezing, nasal congestion, delayed mucociliary clearance, and eustachian tube obstruction after being infected with Rhinovirus 39 (RV39), but there was no statistical difference in the magnitude, frequency, or duration of symptoms between the allergic and nonallergic subjects [78]. Furthermore, total serum IgE levels acutely increased from baseline in RV39 infected AR patients compared to RV39 infected non-AR patients [79]. When observing the effect of RV39 infection on nasal responsiveness to histamine and cold air, allergic subjects experienced twice as much sneezing, rhinorrhea, and secretions prior to RV39 infection, and both subjects with AR and non-AR experienced an increase in sneezing and secretions to intranasal histamine challenge after RV39 infection [80].

## 5. Prevention and Treatment of Allergic Rhinitis

### 5.1. Indoor strategies

For prevention of AR, general allergen and pollution avoidance are recommended when possible [9]. Nasal filters and blockers act as nasal mucosal barriers and can be a useful alternative for some individuals [9]. Additionally, circulating air when indoors is important, as indoor air pollutants can be reduced by improving outdoor and indoor air exchange [9]. In addition to central heating, ventilation, and air conditioning (HVAC) systems, high efficiency particulate air (HEPA) filters can be used to extract 0.3 µm size particles to help reduce allergic triggers [9]. Surgical and N95 masks filter particles with sizes of 3 µm or 0.04 µm, respectively [35]. Extracting inhaled airborne allergens such as pollen, fungal spores, and HDM can significantly decrease IgE-mediated immunologic responses [35]. Hardwood floors, new mattresses and carpets, and bedrooms on higher floors can help reduce HDM concentration and reduce AR [34]. Spending time outdoors in clean air has also been associated with a lower prevalence of AR symptoms [74].

### 5.2. Outdoor Strategies

Multiple studies have shown that exposure to clean air helps decrease the prevalence of AR in children. For example, a retrospective study conducted between 1997 and 2006 found that a decrease in NO_2_, PM, and CO correlated with a yearly decrease in rates of AR [9]. Smoke-free legislation has been adopted in many countries and has been demonstrated to be associated with an improvement in overall child health, although allergic rhinitis was not specifically studied [81].

Air pollution monitoring is essential to help prevent the development and exacerbation of AR in individuals as well. Many countries are now enforcing new policies to help with the prevention of climate change, which in turn will help decrease the onset of AR and aggravation of symptoms. The European Union’s framework for environmental policy has already significantly contributed to diminishing the emissions of air pollutants and improving air quality across Europe [18]. Moreover, environmental performance standards in Europe have been put in place for large combustion plants, limiting emission ranges for NO, NO_2_, SO_2_, PM, and mercury [18]. In the United States, the Clean Air Act has been established to help improve air quality by regulating hazardous air pollutants [9]. Another prevention method that has become more popular recently is shifting from private motorized transport to public transport, cycling, or walking to help reduce greenhouse gases [18].

### 5.3. Phamacotherapeutic Strategies

A variety of treatment options for AR symptoms exist. The most commonly used pharmacotherapy for AR is oral antihistamines with a preference for second-generation antihistamines (e.g., cetirizine, levocetirizine, fexofenadine, loratadine, desloratadine) over first-generation antihistamines (e.g., diphenhydramine, hydroxyzine, chlorpheniramine) because of fewer side effects related to sedation, disrupted sleep, and anticholinergic symptoms [12,82]. Intranasal corticosteroids, which decrease the inflammatory cells and inhibit the release of cytokines, decreasing inflammation of the nasal mucosa, are recommended as the initial treatment for AR, as they have been found to be equally or more effective than oral antihistamines [12,82,83]. Other pharmacologic treatment options for AR include intranasal corticosteroids, intranasal or ocular antihistamines, decongestants, intranasal cromolyn, intranasal anticholinergics, oral leukotriene receptor antagonists, and oral corticosteroids; however, intranasal cromolyn and decongestants are not always suitable for young children [82,83]. AR symptoms may be controlled with monotherapy, but combination therapy is necessary in patients with persistent or severe symptoms [82,83].

Some epidemiological data suggest that the intake of dietary antioxidants may decrease the prevalence of AR [9]. Some studies have suggested that vitamin D supplementation in conjunction with antihistamines may improve treatment response; however, the role of vitamin D in allergic rhinitis has not been definitively established [84,85]. An anti-IgE antibody, omalizumab (Xolair), is effective in reducing nasal symptoms in AR patients as well, although it is not currently approved by the Food and Drug Administration for this indication [83]. The only intervention that can modify AR is specific immunotherapy with aeroallergen extracts (e.g., cat, dog, molds, cockroach, mice, dust mites, pollens, etc.) [11]. Immunotherapy can be considered for patients with moderate or severe AR that is not responsive to typical AR treatments or for patients who wish to avoid or reduce long-term medication use [82]. Targeted immunotherapy consists of patients visiting a physician’s office at regular intervals (typically initially weekly for build-up, then monthly for 3–5 years for maintenance therapy) to receive a small amount of allergen extract subcutaneously or sublingually [83]. Subcutaneous immunotherapy may be more efficacious than sublingual immunotherapy but is less convenient as subcutaneous immunotherapy should be administered in the medical office for monitoring of anaphylaxis, whereas sublingual immunotherapy can be administered at home after the initial dose [83]. A further benefit of allergen immunotherapy in children is that it may prevent asthma onset in children with allergic rhinitis [82].

### 5.4. Knowledge Gaps

Future studies could help advance prevention and treatment of AR in children. First, definitive studies of whether vitamin D supplementation could treat AR are needed as vitamin D is an inexpensive, simple intervention. The development of new treatments to mitigate or counteract the effects of environmental pollutants on the immunologic regulation of AR can also be considered. More importantly, strategies to reduce environmental factors associated with AR need to be more aggressively studied and implemented. Finally, additional studies demonstrating the positive impact that environmental policies have on health, including prevention of AR, may help policy makers make decisions.

## 6. Conclusions

Lifestyle and environmental factors play an important role in the development of allergic rhinitis, one of the most common chronic conditions in the world. Specifically, indoor air pollution, such as HDM, tobacco smoke, and pet dander can significantly increase the onset of AR. Moreover, outdoor air pollution, including TRAP and pollen can increase the risk of AR in children. Preventive measures for AR control through environmental protection policies also have great promise to improve public health. Additionally, viruses may increase the risk and exacerbate the symptoms of AR. The avoidance of these environmental triggers may reduce the development of AR and AR symptoms.
